# 

**Published:** 1911-12

**Authors:** 


### CONTENTS.

ORIGINAL COMMUNICATIONS—

Growing Pains. By L. B. Clarke, M. D., Atlanta, Ga. .................... 461

Cerebral Affections often Mistaken for Neoplasm. Differential Diagnosis of Actual Cases. By Tom A. Williams, MB. CM., Washington, D. C. ........ 465

The Significance of Enlarged Lymph Nodes in Children. By George Kent Varden, M. D., Atlanta, Ga. .................... 468

"606;" Its Administration 455 Times. By Edgar G. Ballenger, M. D., and Omar F. Elder, M. D., Atlanta, Ga. .................... 471

Pin in Bronchus—Report of a Case. By F. G. Hodgson, M. D., Atlanta, Ga. .. 483

Inguinal Hernia Operated on Under Local Anaesthesia. By A. G. Little, M. D., Valdosta, Ga. .................... 484

EDITORIALS .................... 490

SELECTIONS AND ABSTRACTS .................... 491

NEWS AND NOTES .................... 509

BOOK REVIEWS .................... 519

